# Improving the accuracy of heart failure diagnosis in low-resource settings through task sharing and decentralization

**DOI:** 10.1080/16549716.2019.1684070

**Published:** 2019-11-07

**Authors:** Alyssa DeWyer, Amy Scheel, Isaac Omara Otim, Christopher T. Longenecker, Emmy Okello, Isaac Ssinabulya, Stephen Morris, Mark Okwir, William Oyang, Erine Joyce, Betty Nabongo, Craig Sable, Ben Alencherry, Alison Tompsett, Twalib Aliku, Andrea Beaton

**Affiliations:** a Department of Cardiology, Children’s National Health System, Washington, DC, USA; b School of Medicine, Emory University, Atlanta, GA, USA; c Department of Medicine, Lira Regional Referral Hospital, Cardiac Clinic, Lira, Uganda; d Department of Cardiology, University Hospitals Harrington Heart & Vascular Institute, Case Western Reserve University, Cleveland, OH, USA; e Department of Cardiology, Uganda Heart Institute, Mulago Hospital Complex, Kampala, Uganda; f Department of Cardiology, Cincinnati Children’s Hospital Medical Center, Cincinnati, Ohio, USA; g Department of Pediatrics, University of Cincinnati College of Medicine, Cincinnati, OH, USA

**Keywords:** Echocardiography, Uganda, task-shifting, telemedicine, training

## Abstract

**Background**: Task sharing of TTE may improve capacity for heart failure diagnosis and management in patients in remote, low-resource settings but the impact on diagnostic accuracy and patient outcomes has not been studied.

**Objectives**: Determine feasibility and impact of non-expert training in transthoracic echocardiography (TTE) to improve the diagnosis and outcomes of patients with suspected heart failure in Uganda.

**Methods**: This two-part study examined an innovative training program to develop TTE competency among non-experts and used a pre-post design to determine the impact of decentralized TTE. Four of 8 non-experts (50%) passed a three-part training course. The training comprised of distance learning through a web-based curriculum, a 2-day hands-on workshop with cardiologists, and independent practice with remote mentorship. Continuous measures were compared (pre- vs. post-TTE) using t-tests or Wilcoxon rank-sum tests as distributionally appropriate and categorical variables assessed through chi-square testing. Sensitivity and specificity were calculated according to standard methodology comparing diagnosis pre- and post-TTE during phase 2.

**Results**: Performance in the post-training phase showed good agreement with expert categorization (κ = 0.80) with diagnostic concordance in 421 of 454 studies (92.7%). TTE changed the preliminary diagnosis in 81% of patients, showing low specificity of clinical decision-making alone (14.2%; 95% CI 10.1–19.2%). Dilated cardiomyopathy, hypertensive heart disease with preserved systolic function, and right heart failure were the most underdiagnosed conditions prior to TTE while hypertensive heart disease with decreased systolic function was the most over-diagnosed condition.

**Conclusions**: In conclusion, non-expert providers can achieve a high level of proficiency for the categorization of heart failure using handheld TTE in low-resource settings and use of telemedicine and remote mentorship may improve performance and feasibility. The addition of TTE resulted in substantial improvement in etiological specificity. Further study is needed to understand implications of this strategy on healthcare utilization, long-term patient outcomes, and cost.

## Background

Heart failure has been described as a global pandemic [1] affecting over 26 million people worldwide [2]. While the incidence and prevalence of heart failure remain poorly characterized in sub-Saharan Africa [3], the 2017 Global Burden of Disease study reported 11.7–15.1% of deaths in this region were attributable to cardiovascular disease [2]. Transthoracic echocardiography (TTE) is known to improve the sensitivity and specificity of heart failure diagnosis and is recommended as best practice by the American Heart Association and the American College of Cardiology [4]. The clinical guidelines of many other countries, including Uganda, also recommend hospital-based TTE, but TTE is rarely available outside of urban centers [5,6]. Task sharing of TTE with non-expert providers may improve capacity for heart failure diagnosis and management in patients in remote, low-resource settings [6], but the impact of a task-sharing model on diagnostic accuracy has not been studied. The objectives of this study were two-fold: (1) To examine the efficacy of a mixed-methods TTE training program for non-experts, relying on handheld TTE technology and telemedicine to support limited hands-on training, and (2) To determine the impact of decentralized TTE to improve the diagnosis of patients with heart failure in a remote, resource-limited setting.

## Methods

The mission of the Uganda Heart Institute includes strengthening of regional centers for decentralized cardiovascular care. The strategy of this program has included regular visits to district and regional referral hospitals for patient consultation and ongoing provider education. However, these outreach clinics are limited by both financial constraints and the large patient volumes at the main tertiary center limiting on-site staff to provide ongoing care and timely diagnosis between clinics.

**Figure 1.**
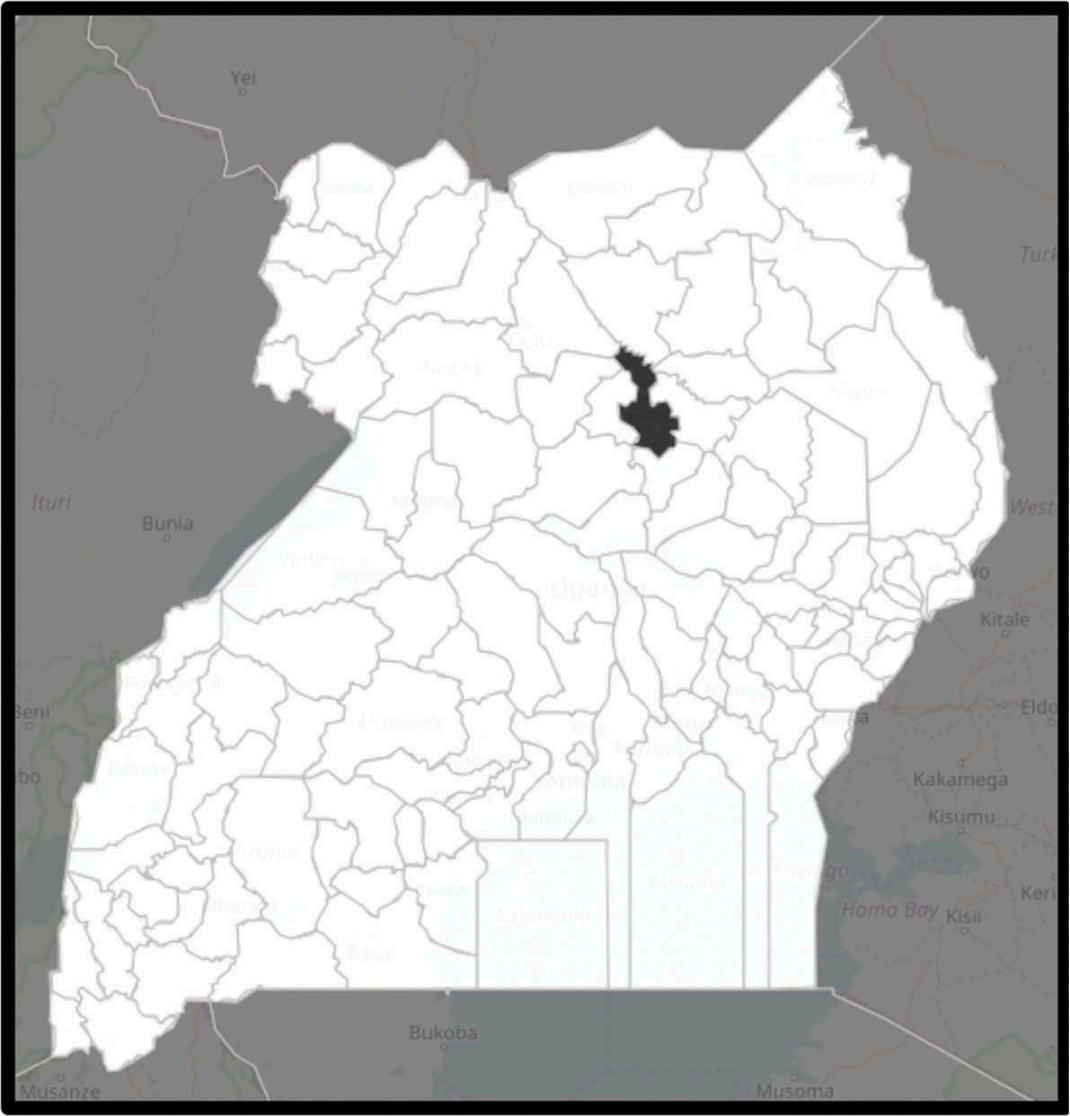
Lira district, Northern Uganda. This study took place at Lira Regional Referral Hospital, serving eight surrounding districts and approximately 2,000,000 persons.

A literature review and discussion with local providers identified heart failure as the most common cardiovascular diagnosis in the region, as well as the condition that posed the greatest diagnostic challenge [7–9]. In 2015, through partnership with RHDAction (global movement to reduce the burden of rheumatic heart disease (RHD), RHDAction.org), an innovative pilot program of task sharing was developed to facilitate decentralized diagnosis of heart failure. A single regional referral hospital in Northern Uganda, Lira Regional Referral Hospital (LRRH), serving eight districts and approximately 2,000,000 persons [10] was selected for the program given its remote location with a large catchment area (Figure 1). No TTE was available at LRRH prior to study commencement.

**Figure 2.**
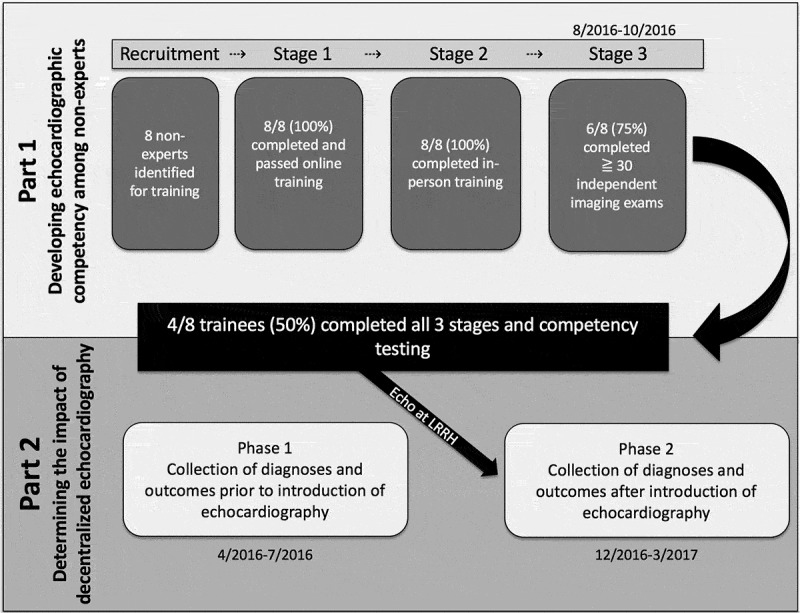
Study flow diagram. The study consisted of two parts: part 1 focused on developing TTE competency among non-experts and part 2 focused on determining the impact of decentralized TTE.

The study consisted of two parts: part 1 focused on developing TTE competency among non-experts (Table 1, Figure 2) through use of GE Vscan V.1.2 (GE Medical Systems, Milwaukee, Wisconsin, USA) and part 2 focused on determining the impact of decentralized TTE (Figure 2). The methods for this study are described in brief below, with detailed methods available in the supplemental appendix.

**Table 1.** Three-stage program to develop TTE competency among non-experts.

Stage	Format	Details	Assessment
Stage 1	Distance Learning	Free 14-part web-based curriculum (WiRED*)	Project-specific 20-question quiz (REDCap(12)); >80% correct
Stage 2	Hands-on Training	2-day workshop with cardiologists, focus on cardiac anatomy, physiology, TTE physics, standard views (VScan^†^) Table 2(a), and interpretation (Table 2(b)) 8 hours hands-on scanning	Complete workshop
Stage 3	Independent practice w/remote mentorship	10 week practicum, TTE on all patients at LRRH presenting with suspected heart disease, uploaded images to DropBox, TTE interpretation to REDCap(12), reviewed in 24 hours by US/Ugandan cardiologist with feedback through REDCap(12) – image quality, interpretation.	Log minimum of 30 studies
Competency Assessment	10 Patients + 10 Computer-based cases	Mix of normal and known pathology, quality and completeness of TTE and interpretation to one of 8 diagnostic categories (normal, pericardial disease, hypertensive heart disease with preserved systolic function, hypertensive heart disease with decreased systolic function, dilated cardiomyopathy, valvular heart disease, right heart failure, other heart disease)	>80% for image acquisition and >80% for diagnostic classification

*http://www.wiredhealthresources.net/EchoProject/, ^†^Vscan, General Electric Medical Systems, Milwaukee, Wisconsin, USA

**Table 2.** Comparison of agreement and accuracy between non-experts and experts (n = 454), 2(a) heart failure diagnosis and 2(b) Component diagnoses.

	n (%)	k (95%CI)	Sensitivity (%, 95% CI)	Specificity (%, 95% CI)	PPV (95% CI)	NPV (95% CI)
2A (n = 454)						
Overall	421 (92.7%)	0.80 (0.73–0.87)	n/a	n/a	n/a	n/a
Normal	96 (21.1%)	0.97 (0.94–0.99)	94.8 (88.3–98.3)	97.8 (95.6–99.0)	91.9 (85.1–95.8)	98.6 (96.8–99.4)
HHD w/preserved systolicfunction	45(9.9%)	0.95 (0.90–1.0)	91.1 (78.8–97.5)	98.5 (96.8–99.5)	87.2 (75.4–93.8)	99.0 (97.5–99.6)
HHD w/decreased systolicfunction	126(27.8%)	0.98 (0.92–0.98)	93.7 (87.9–97.2)	99.1 (97.4–99.8)	97.5 (92.7–99.2)	97.6 (95.4–98.8)
DCM	78 (17.2%)	0.96 (0.93–0.99)	93.6 (85.7–97.9)	97.3 (95.2–98.7)	88.0 (79.8–93.1)	98.7 (96.9–99.4)
VHD	45 (9.9%)	0.94 (0.88–0.99)	88.9 (75.9–96.3)	99.8 (98.7–99.9)	97.6 (84.9–99.7)	98.8 (97.3–99.5)
PE	9 (2.0%)	1	100 (66.4–100.0)	100 (99.2–100.0)	100	100
RHF	21 (4.6%)	0.95 (0.88–1.0)	90.5 (69.6–98.8)	99.5 (98.3–99.9)	90.5 (70.3–97.4)	99.5 (98.3–99.9)
Other	34 (7.5%)	0.93 (0.87–0.99)	88.2 (72.5–96.7)	100.0 (99.1–100.0)	100.0	99.1 (97.8–99.8)
2B (n = 454)						
Severely Dilated LV	55 (11.9)	0.63 (0.53–0.75)	74.6 (61.0–85.3)	94.1 (91.3–96.2)	63.1 (52.9–72.2)	96.5 (94.6–97.7)
Severely Reduced LV function	84 (18.2%)	0.66 (0.57–0.75)	73.8 (63.1–82.8)	93.4 (91.4–95.7)	71.3 (62.5–78.7)	94.1 (91.8–95.8)
Severely Dilated RV	73 (15.8)	0.43 (0.31–0.55)	52.1 (40.0–63.9)	91.0 (87.7–93.6)	52.1 (42.5–61.5)	91.0 (85.5–91.5)
Severely Reduced RV function	99 (21.5)	0.66 (0.57–0.75)	70.7 (60.7–79.4)	93.7 (90.6–95.9)	75.3 (66.8–82.2)	92.1 (85.5–91.5)
Moderate/Severe Mitral regurgitation	41 (8.9)	0.62 (0.51–0.73)	100 (91.4–100.0)	90.2 (87.0–92.9)	50.0 (42.8–57.2)	100
Mitral stenosis	29 (6.3%)	0.42 (0.17–0.66)	27.6 (12.7–47.2)	100.0 (99.2–100.0)	100	95.4 (94.3–96.3)
Moderate/Severe Aortic regurgitation	4 (0.9)	0.21 (0.00–0.54)	75.0 (19.4–99.4)	95.6 (93.3–97.3)	13.0 (6.9–23.4)	99.8 (98.9–100.0)
Large Pericardial effusion	6 (1.3%)	0.36 (0.06–0.66)	83.3 (35.9–99.6)	96.5 (94.4–98.0)	23.8 (14.6–36.3)	99.8 (98.7–100.0)
Dilated IVC	102 (22.1)	0.57 (0.47–0.67)	56.9 (46.7–66.6)	95.0 (92.2–97.0)	76.3 (66.6–83.9)	88.6 (86.1–90.7)
Severely Thickened IVS	21 (4.6)	0.33 (0.16–0.49)	81.0 (58.1–94.6)	88.0 (84.5–90.9)	24.3 (18.8–30.8)	99.0 (97.6–99.6)

CI: Confidence Interval, HHD: Hypertensive heart disease, DCM: Dilated Cardiomyopathy, VHD: Valvular heart disease, PE: Pericardial effusion, RHF: Right heart failure, LV: Left ventricle, RV: Right ventricle, IVC: Inferior vena cava, IVS: Intraventricular septum.

### Patient recruitment

Patients with suspicion of heart failure were recruited from clinical referrals from the outpatient/acute care and inpatient wards. An attempt was made to broadly capture all patients ≥ 1 year of age presenting with signs or symptoms of possible heart failure. Inclusion criteria focused on signs/symptoms suspicious of heart failure developed by consensus between the LRRH team and investigators. The intent was to improve diagnostic accuracy; thus, criteria thought to be highly sensitive, with a trade-off of lower specificity were included and any one of the following was acceptable: (1) Dyspnea or exercise intolerance > 1 month in duration, (2) Lower extremity edema, (3) Abdominal distention believed to be ascites, (4) Cough > 1 month or wheezing < 1 month & Chest X-ray not consistent with focal lung infection or TB, (5) Tachycardia not attributed to infection/fever. Defined as >100 bpm in adults, >120 bpm for children ≥ 5 years, >140 bpm for children <5 years, (6) Cyanosis or clubbing, (7) Syncope, (8) Chest pain or palpitations, with two or more of the following (HTN, DM, smoking, age >50), (9) Acute stroke (i.e. the reason for clinical presentation was a stroke syndrome), and (10) High provider suspicion of cardiovascular pathology based on diagnostic workup (e.g. cardiac murmur, displaced PMI, elevated JVP, EKG or chest radiographic finding).

All frontline healthcare providers were educated about the inclusion criteria and research staff rounded to help with patient identification.

#### Part 1: developing TTE competency among non-experts

Eight non-expert health professionals working at LRRH and without previous experience in TTE – including three nurses, two clinical officers, one sonographer, and two physicians (one medical officer and one internal medicine specialist) – were recruited to participate based on expressed interest in the program, availability, input from hospital leadership, and plans to continue working at LRRH for the duration of the study. These health professionals gave written informed consent for participation. Training consisted of four stages (Table 1, Figure 2, details in supplemental appendix): 1) completion of a web-based, freely available ultrasound curriculum (http://www.wiredhealthresources.net/EchoProject/), 2) completion of a 2-day echocardiography course led by four expert cardiologists, 3) independent practice with remote mentorship, targeting acquisition and interpretation of least 30 echocardiographic studies, 4) Competency assessment.

Participants who passed all three stages of training and the competency assessment were utilized to obtain and interpret TTEs at LRRH as Part 2 of this study. Real-time expert interpretation was not utilized during Part 2, but all TTEs were retrospectively reviewed in a blinded fashion after study completion and categorized for determination of non-expert accuracy.

#### Part 2: impact of decentralized TTE on diagnostic accuracy

Part 2 was a pre-post study design conducted between April 2016 and April 2017 prior to and after the introduction of focused TTE. Demographic data (age, sex), clinical presentation and data (area of service, inclusion criteria, pre-existing relevant health conditions, prior cardiovascular medications, vital signs at presentation), diagnostic classification (heart failure yes/no, final diagnosis), and outcome data (length of stay, outcome, referrals) were collected on patients meeting inclusion criteria utilizing standard data capture forms.

#### Part 2: pre-intervention survey

A 4-month pre-intervention survey was undertaken prior to TTE introduction to understand the baseline diagnostic profile, referral practices, and outcomes of patients with suspected heart failure.

After local consultation and records review for the most prevalent causes of patient heart failure, we identified the major diagnostic categories at presentation as normal, pericardial disease, hypertensive heart disease with preserved systolic function (thickened IVS without systolic dysfunction), hypertensive heart disease with systolic dysfunction (thickened IVS with systolic dysfunction), dilated cardiomyopathy (all cause), valvular heart disease (rheumatic or other), right-sided heart failure and other (to include congenital heart disease and primary arrhythmia among others). A simplified diagnostic TTE protocol as outlined above (Supplemental Table 1A/1B), and a 12-lead ECG was added to the existing practice of history, physical exam, and occasional ancillary testing (such as chest radiography). ECG interpretation was supported through a 1 day in-person training on ECG basics but was mainly limited to the automatic read and rhythm diagnosis.

#### Phase 2: TTE phase

A 4-month post-intervention survey was undertaken with TTE integrated into the diagnostic workup for those with suspected heart failure, utilizing parallel recruitment, inclusion criteria, and case report forms. In addition, additional pre- and post-TTE data were collected from providers (preliminary diagnosis before TTE, clinician impression of heart failure pre-TTE yes/no, do TTE findings explain patient symptoms, clinician impression of heart failure post-TTE yes/no).

Focused TTE and ECG by trained non-experts (Part 1) were performed as part of the clinical assessment and used to aid in final diagnostic classification. Consultation with other clinicians in this study was neither encouraged nor discouraged as this phase was a pragmatic application of TTE into clinical care. Data were not captured on rates of consultation within the group. Real-time expert interpretation was not utilized during this stage, but all TTEs were retrospectively reviewed by cardiologists blinded to on-ground diagnosis after study completion and categorized for determination of non-expert accuracy.

### Data management and statistical analysis

Study data were collected and managed using REDCap (Research Electronic Data Capture) tool, a secure, web-based application designed to support data capture for research studies, hosted at Children’s National Medical Center, Washington DC [11]. All data were analysed using MedCalc for Windows Version 18.2.1 (MedCalc Software, Ostend, Belgium).

#### Part 1

The number of TTEs performed by each participant was captured. Results of competency testing were reported as pass (≥80%) or fail (<80%). Non-expert performance post-training were compared with remote expert interpretations. TTE studies were uploaded onto google drive and were downloaded off the server by the remote experts. Remote experts entered interpretation into RedCap and the treating clinicians downloaded the reports off the RedCap server. Discordance between on-site and remote readings was considered with differences in final category of heart failure, including no heart failure. Kappa coefficient (κ) was calculated as the degree of agreement between the two. Sensitivity, specificity, and predictive values were determined from the percentage of patients with true-positive and true-negative results with 95% CIs by heart failure category. Additionally, data were also analysed for agreement on the echocardiographic components of diagnosis. Continuous data are reported as mean ± SD or as median and IQRs if not normally distributed, and categorical data are reported as numbers and percentages.

#### Part 2

Demographic, clinical, diagnostic, and outcomes data were collected on standardized paper reporting forms and transcribed into REDCap. Data are presented by study phase as mean and standard deviation, median and interquartile range, and number and percentage as appropriate. Continuous measures were compared (pre- vs. post-TTE) using t-tests or Wilcoxon rank-sum tests as distributionally appropriate and categorical variables assessed through chi-square testing. Sensitivity and specificity were calculated according to standard methodology comparing diagnosis pre- and post-TTE during phase 2.

#### Ethical approval

The study was approved by the institutional review boards of Makerere University School of Medicine, the Uganda National Council of Science and Technology, University Hospitals Cleveland Medical Center (Cleveland, Ohio, USA) and Children’s National Medical Center (Washington, DC, USA).

## Results

### Part 1

Eight non-expert participants began the TTE-training course. Of these, all eight passed the competency assessment at the end of Stage 1 with an overall mean score of 86.7%, and all eight were present for the duration of Stage 2 training. Only six of the eight participants completed the minimum of 30 practice TTE studies during Stage 3 and were eligible for final competency testing. Of the six participants who took the Stage 4 final competency assessment, four received passing scores of ≥80% and moved into the post-training phase as sonographers for Part 2, including one nurse, one medical officer, one sonographer and one internal medicine specialist.

A total of 455 TTEs were performed during the post-training phase, with one being excluded from this analysis secondary to image loss. The average time to review a study was 5 min. Image quality was considered good (full imaging protocol achieved and able to analyze without imaging deficits) in 82%, acceptable (full imaging protocol achieved, but up to two parameters not able to be fully assessed) in 12%, poor (missing frames from imaging protocol and/or >2 parameters not able to be fully assessed) in 6% with no studies categorized as unable to interpret.

Overall agreement during the post-training phase between non-expert and expert heart failure categorization was good (κ = 0.80, 95% CI 0.73–0.87), coinciding in 421 (92.7%) of 454 studies (Table 2(a)). Non-expert and expert agreement were best for the diagnoses of pericardial effusion (κ = 1.0) but all categories showed very good agreement. Of the 96 studies considered normal by non-experts, only eight (8.3%) had significant pathology that was missed: dilated cardiomyopathy (4), valvular heart disease (1), right heart failure (2), and other (congenital heart disease) (1). (Table 2(a)).

Concordance among component diagnoses was worse than the concordance among heart failure diagnoses (κ ranging from 0.21 to 0.66; Table 2(b)). Non-expert and expert agreement were best for reduced right and left ventricular function (both with κ = 0.66) (Table 2(b)).

### Part 2

The clinical characteristics of the study population are summarized in Table 3. Between phase 1 and phase 2, an acute care/emergency ward was opened at LRRH. Prior to the opening of this unit, patients waited in outpatient department lines, we introduced a triage system to try to prioritize more acutely ill patients. There were more referrals from the combined outpatient/acute care area compared to the inpatient ward in phase 2 (p = 0.012). As expected in an observational study, there were also many differences in inclusion criteria, pre-existing health conditions, and prior cardiovascular diagnoses between phases (Table 3).

**Table 3.** Demographics of enrolled patients in Phase 1 (pre-TTE) compared to Phase 2 (post-TTE).

		Phase 1(n = 424)	Phase 2(n = 454)	p-value
Age (Median, IQR)		58 (39–70)	59 (38–70)	0.84
Gender (% female)		254 (59.9%)	272 (59.9%)	0.13
CLINICAL DATA				
Area of Service	Outpatient/Acute Care	281 (66.3)	337 (74.2)	**0.01**
	Inpatient Ward	143 (33.7)	117 (25.8)	
Inclusion Criteria	Dyspnea or exercise intolerance > 1 month	317 (74.8)	422 (92.3)	**<0.001**
	Lower extremity edema	224 (52.8)	218 (48.0)	0.16
	Abdominal distention believed to be ascites	76 (17.9)	57 (12.6)	**0.03**
	Cough > 1 month or wheezing < 1 mo, and CXR not consistent with focal lung infection or TB	27 (6.3)	78 (17.2)	**<0.001**
	Tachycardia not attributed to infection	112(26.4)	94 (20.1)	**0.03**
	Cyanosis or clubbing	1 (0.2)	3 (0.7)	0.27
	Syncope	9 (2.1)	17 (3.7)	0.16
	Chest pain or palpitations, with 2 or more of the following (HTN, DM, smoking, age >50)	220 (51.9)	203 (44.7)	**0.03**
	Acute stroke	30 (7.1)	33 (7.3)	0.9
	High provider suspicion of cardiovascular pathology	9 (2.1)	0 (0.0)	**0.002**
	Patients meeting ≥3 inclusion criterion	193 (45.5)	199 (43.9)	0.63
Pre-existing Health Conditions	History of Hypertension	244 (57.5)	214 (47.1)	**0.002**
	Diabetes	70 (16.5)	35 (7.7)	**<0.001**
	Chronic Kidney Disease	6 (1.4)	0 (0.0)	**0.01**
	Smoker	43 (10.1)	24 (5.3)	**0.008**
	Heavy or Chronic Alcohol Use	100 (23.6)	114 (25.1)	0.61
	HIV	28(6.6)	45 (9.9)	**0.08**
Prior Cardiovascular Diagnosis	Pericardial Disease	2 (0.5%)	5 (1.1%)	0.32
	Hypertensive Heart Failure	77 (18.2%)	25 (5.5%)	**<0.001**
	Congenital Heart Disease	1 (0.02%)	1 (0.02)	1
	Rheumatic or other valvular heart disease	24 (5.7%)	12 (2.6%)	**0.02**
	Right heart failure	0	2 (0.4%)	0.19
	Dilated cardiomyopathy	0	6 (1.3%)	**0.01**
	Arrythmia	9 (2.1%)	10 (2.2%)	0.92
	Other	26 (6.1%)	12 (2.6%)	**0.01**
Cardiovascular Medication Prior to Visit	Any	232 (54.7)	170 (37.4)	**p < 0.001**
Vitals at Presentation	Febrile (*n* total = 315, 454)	11 (3.5)	3 (0.7)	**0.004**
	Pre-Hypertensive (*n* total = 405, 450)	92 (22.7)	94 (20.9)	0.52
	Hypertensive Stage 1(*n* total = 405, 450)	80 (19.8)	103 (22.9)	0.26
	Hypertensive Stage 2(n total = 405, 450)	151 (37.2)	144 (32)	0.11
	Bradycardia	31 (7.3)	35 (7.7)	0.82
	Tachycardia	121 (28.5)	126 (27.8)	0.82
	Tachypnea (RR >25)	92 (21.7)	76 (16.7)	0.06
	Hypoxemia (Sat < 95%) (n total = 291, 451)	114 (39.2)	215 (47.7)	**0.01**

### Phase 1 results

There were 424 patients captured during the pre-intervention period (Phase 1). Of these, the most common diagnoses included hypertensive heart failure (52.6%), no heart failure (19.8%) and heart failure not otherwise specified (NOS) (16.0%). Almost one-third of the patients (n = 139, 32.8%) were admitted to the inpatient wards at presentation with a median length of stay of 4 days (IQR), and 27 (6.4% of the total population) died during that admission.

### Phase 2 results

There were 454 patients captured during the post-intervention, phase 2. Of these the most common final diagnoses included hypertensive heart disease with reduced systolic function (26.4%), dilated cardiomyopathy (17.6%), and no heart failure (15.4%). One hundred and seventy-three patients (38.1%) were admitted to the inpatient wards with a median length of stay of 5 days (IQR), and 21 (4.6%) died during that admission.

#### The impact of TTE on diagnostic accuracy

In Phase 2, TTE evaluation resulted in a change between preliminary (clinical) and final (clinical + TTE) major diagnostic category in 367 (80.8%) patients. Incorporating TTE into diagnostic decision-making during phase 2, clinical impression had a reasonable sensitivity of 89.9% (95% CI 85.0–93.6%) but a very low specificity of 14.2% (95% CI 10.1–19.2%).

**Figure 3.**
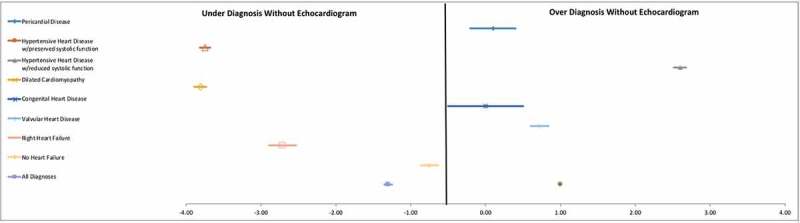
Forest plot of diagnostic accuracy. Introduction of TTE improved the specificity of heart failure diagnosis, showing with clinical impression alone dilated cardiomyopathy, hypertensive heart disease with preserved systolic function, and right heart failure were underdiagnosed and hypertensive heart disease with decreased systolic function was over-diagnosed.

In the study population, this led to a clinical positive predictive value for heart failure without use of TTE as 47.0% (95% CI 45.3–48.7), and a clinical negative predictive value for heart failure of 62.5% (95% CI 50.1–73.5). Dilated cardiomyopathy, hypertensive heart disease with preserved systolic function, and right heart failure were the most underdiagnosed conditions prior to TTE while hypertensive heart disease with decreased systolic function was the most over-diagnosed condition (Figure 3).

**Figure 4.**
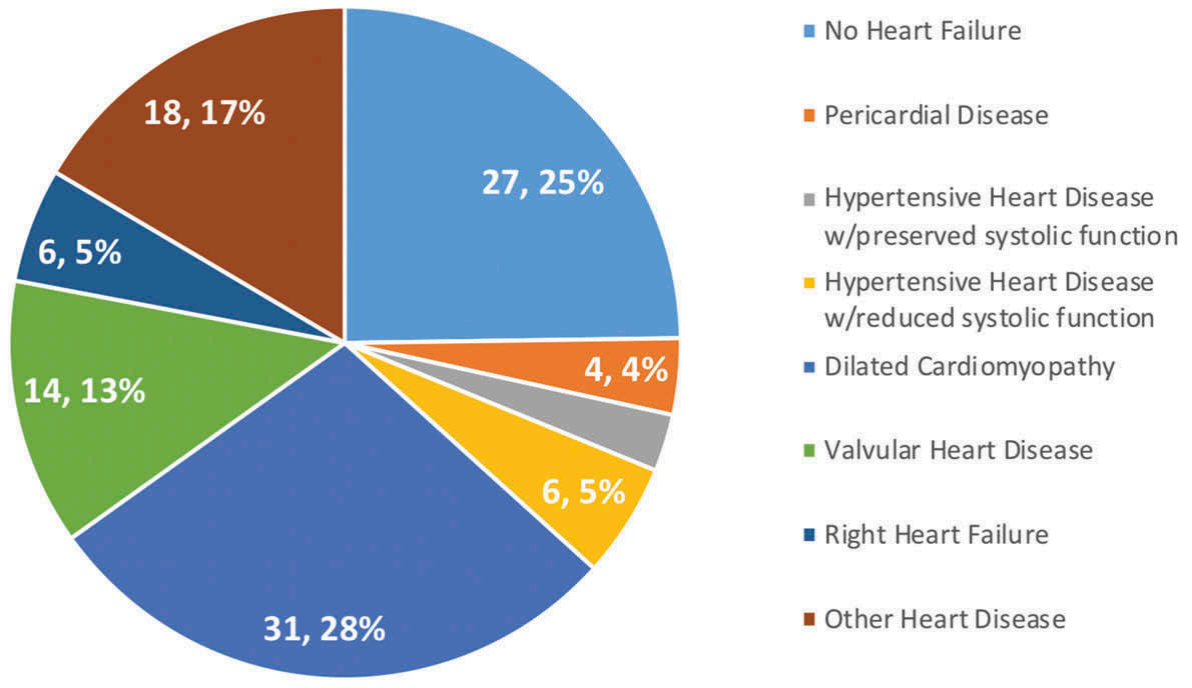
Heart failure NOS diagnoses. Prior to TTE, heart failure NOS was a common diagnostic category. TTE eliminated this category, revealing the etiology of symptoms, including those not in heart failure, in all cases.

In addition, access to TTE allowed for elimination of the diagnostic category heart failure NOS, the preliminary diagnosis of 68 patients (16%) of patients in Phase 1 and 109 patients (24%) in Phase 2. In Phase 2, TTE revealed that 25% of these patients were ultimately found to have no evidence of heart failure allowing for continued work-up of differential diagnosis. Of the 75% with heart disease, the majority had dilated cardiomyopathy, valvular heart disease, and other heart diseases (arrhythmia, congenital, among others) (Figure 4). ECG provided additional diagnostic benefit for 33 patients (7.3%); 25 with atrial fibrillation, 4 with atrial flutter, 3 with complete heart block, and 1 with supraventricular tachycardia.

In phase 2, there were more patients who were discharged to home without follow-up (p < 0.01) and less patients scheduled for follow-up at the outpatient cardiac clinic (p < 0.01). Phase 2 also saw fewer patients self-discharging from the inpatient wards against medical advice. There was no difference in death during hospitalization between phases, though total numbers were small and lacked sufficient power (p = 0.24) (Table 4).

**Table 4.** Comparison of outcomes between Phase 1 (pre-TTE) and Phase 2 (post-TTE).

	Phase 1(n = 424)	Phase 2(n = 454)	p-value
Discharge to home	105 (24.8%)	179 (39.4%)	**<0.01**
Self-Discharge (against medical advice)	9 (2.1%)	2 (0.4%)	**0.02**
OPD Follow-up Scheduled	267 (73.0%)	225 (49.6%)	**<0.01**
Transfer to another facility	16 (3.8%)	27 (5.9%)	0.15
Death during inpatient hospitalization	27 (6.4%)	21 (4.6%)	0.24

## Discussion

Heart failure is a major cause of cardiovascular morbidity and mortality in sub-Saharan Africa [6,12]. This study demonstrated that limited hands-on training coupled with telemedicine and web-based support can provide excellent training for non-experts in basic heart failure diagnosis using handheld TTE. This represents a significant innovation as current capacity to diagnose and treat heart failure in this region is extremely limited outside of tertiary centers [5]. Our study also demonstrates that the addition of TTE dramatically improves the specificity of heart failure diagnosis and eliminates the category of heart failure with unknown cause (NOS), which accounted for more than one-quarter of patients with suspected heart failure prior to TTE.

These findings have broad implications for supporting decentralized cardiac care in resource-limited settings. Despite the current Ugandan guidelines recommending chest x-ray, ECG, and TTE as standard investigations for suspected heart failure [13], only 3% of the hospitals in Uganda (including health center IV’s, regional referral hospitals and national referral hospitals) have both equipment and trained staff in all of these imaging modalities [14]. Use of handheld TTE may provide a more cost-effective and convenient option for equipment in these facilities. The technical adequacy of handheld TTE has been supported by a prior study in Rwanda, suggesting, as we found, that the functional limitations of handheld TTE (lack of spectral Doppler and M-mode imaging) did not impair the broad categorization of heart failure diagnosis [6].

The training used in this study represents an innovation that could potentially facilitate the broader and more consistent training and competency testing needed to ensure high-quality care. Graduates of our training program showed very high agreement (93%, K = 0.80) between non-expert and expert categorization of heart failure, and 82% of the TTE studies were classified as good quality. These results represent substantial improvement from a previous study, which utilized only a 4-day training (similar to our Stage 2) of family physicians in remote Spanish primary care clinics, resulting in only 58% agreement of heart failure categorization and 35.4% of the studies classified as good quality [15]. The practicum period included in our training design facilitated ongoing remote mentorship and skill-building, which are likely responsible for this high performance. Additionally, in this study, we utilized both national and international expert mentorship, demonstrating the feasibility of telemedicine support provided within country from a tertiary cardiac center. The small file size of the TTE studies performed on the device used in this study provides an additional advantage for seamless uploading and downloading of images for telemedicine.

The practicum period also served as an informal measure of non-expert investment, availability, and skill level. Of the eight non-experts who were originally recruited for the project, only six completed the minimum number of practicum studies needed to advance and only four passed the final competency test to advance as a program sonographer. The lack of trainee advancement in the program was primarily due to the high ratio of patients to staff at LRRH and a lack of time to take on additional responsibilities. Specific issues included trainees that worked at other medical sites in combination with LRRH had less time to complete the training due to other work commitments, some trainees had staffing shortages in their departments and had difficulty finding time to scan, and physicians were also more likely to travel for meetings or regional conferences, therefore, unable to complete the required about of scans. Our experience suggests that task shifting of TTE might be best accomplished when including some degree of both self-selection and rigorous skill assessment.

The largest impact of adding TTE was, unsurprisingly, the improvement in specificity of the etiology of heart failure. While pericardial disease and congenital heart disease were diagnosed with high accuracy without the addition of TTE, dilated cardiomyopathy and right heart failure were significantly under-diagnosed and hypertensive heart disease with reduced systolic function was over-diagnosed. TTE was able to differentiate between those with intraventricular septal hypertrophy and preserved systolic function (i.e. hypertensive heart disease with preserved systolic function) and those with intraventricular septal hypertrophy with diminished LV function (i.e. hypertensive heart disease with reduced systolic function). Even though our study was not designed to assess appropriateness of therapeutics, knowing the correct etiology of heart failure symptoms likely leads to more targeted therapeutics, which may improve long-term costs, patient symptoms, and outcomes.

While our study confirms that a decentralized strategy of heart failure diagnosis unequivocally improved the quality of patient diagnosis, we failed to demonstrate differences in mortality with the addition of TTE. This could have resulted from low numbers of death, the limited ability to assess longer-term mortality (only inpatient outcome), and the fact that many who succumb to inpatient mortality at the time of diagnosis present with advanced, decompensated heart failure. A broader study is needed to understand if more specific diagnoses in milder cases lead to less hospitalizations and improved survival as well as contribute to more appropriate healthcare utilization.

Our study has several limitations. First, we did not mandate that in the post-training period final non-expert diagnosis be made in isolation. While we did not capture data on collaboration, it was common for two or more of the sonographers to review images together, necessarily increasing accuracy. However, collaboration between imagers is common in the clinical setting and, intentionally, we did not limit it in this pragmatic study. Second, our study focused only on the diagnosis of definite heart failure, and we did not train or evaluate the ability of our non-experts to diagnose more subtle forms of heart disease, which are likely common in this low-resource community. Third, in an effort to simplify training and broadly classify heart failure patients into wide diagnostic categories, we utilized only qualitative and semi-quantitative diagnostics. In this structure, more complete evaluation by a cardiologist with fully functional TTE equipment would be needed in cases where surgical or catheter interventions were being considered.

In conclusion, this study demonstrates that non-expert providers can achieve a high level of proficiency for the categorization of heart failure using handheld TTE in low-resource settings. In addition, we have shown that a model incorporating low-bandwidth telemedicine and web-based reporting can facilitate a longer mentorship period, resulting in high performance. The largest advantage seen after the initiation of TTE was substantial improvement in etiological specificity. Further study is needed to understand implications of this strategy on healthcare utilization, long-term patient outcomes, and cost. The application of this strategy has the potential to significantly improve task shifting of TTE in resource limited settings, improving the diagnosis and care for heart failure patients around the globe.

## Paper context

A single study (Kwan, 2013) suggests task shifting of echocardiography to non-experts may improve diagnostic capacity for heart failure, but there is no data on the training, competency testing, or accuracy of non-experts. This manuscript establishes a successful limited hands-on training program through telemedicine and web-based support, and provides strong justification for how a model incorporating low-bandwidth telemedicine and web-based reporting can facilitate a longer mentorship period, resulting in high performance.
