# Supplementary material for: Improving the accuracy of heart failure diagnosis in low-resource settings through task sharing and decentralization
Source: Glob Health Action. 2019 Nov 7;12(1):1684070. doi: 10.1080/16549716.2019.1684070 (PMC6844369; doi:10.1080/16549716.2019.1684070)
Supplement: Supplemental Material [file ZGHA_A_1684070_SM4654.zip › Appendix Table 1A_B.docx]

Supplementary Table

Table 1A: Echo

cardiographic

Acquisition Protocol

Parasternal Long Axis

2D

Left and right ventricular size and function, mitral and aortic valve

morphology, pericardial effusion, ventricular septal thickness

Color Doppler

Mitral and aortic valve regurgitation/stenosis

Parasternal Short Axis

2D

Aortic valve morphology, left

and right ventricular size and function

Color Doppler

Aortic valve regurgitation/stenosis

Apical 4/5 Chamber

2D

Left and right ventricular size and function, mitral and tricuspid morphology,

pericardial effusion

Color Doppler

Mitral and tricuspid valve

regurgitation/stenosis

Subcostal

2D

IVC size

Table 1B: Echocardiographic Interpretation Parameters

Component

Assessment

Categories

Left Ventricular Systolic

Function

Visual inspection

Normal, mildly reduced, severely reduced

Left Ventricular

Size

Visual inspection

Normal, mildly enlarged, severely enlarged

Right Ventricular Systolic

Function

Visual inspection

Normal, mildly reduced, severely reduced

Right Ventricular Size

Visual inspection

Normal, mildly enlarged,

severely enlarged

Intraventricular Septal

Thickness

Semi

-

Quantitative

Normal (<1cm), mild/moderate (1

-

1.7cm), and

severe (

≥

1.8cm)

Valvular Regurgitation

Visual Inspection

None, Mild, Moderate, Severe

Mitral Stenosis

Visual Inspection

None, Present

Pericardial Effusion

Semi

-

Quantitative

None, Small (<0.5cm), Large (>0.5cm)

Inferior Vena Cava Size

Semi

-

Quantitative

Normal, Dilated (>1.5cm)
