# Supplementary material for: Improving the accuracy of heart failure diagnosis in low-resource settings through task sharing and decentralization
Source: Glob Health Action. 2019 Nov 7;12(1):1684070. doi: 10.1080/16549716.2019.1684070 (PMC6844369; doi:10.1080/16549716.2019.1684070)
Supplement: Supplemental Material [file ZGHA_A_1684070_SM4654.zip › LIRA Appendix 1- Part 1 Detailed Methods.docx]

Appendix A: Part 1 Detailed Methods

**METHODS**

**Part 1: Developing TTE Competency Among Non-Experts: Intervention and Training**

Part 1 TTE training consisted of four stages.

Part 1, Stage 1: In the first stage, each participant was asked to independently complete a freely available 14-part, web-based ultrasound training curriculum: the Wired modules (<http://www.wiredhealthresources.net/EchoProject/>). Wired modules are targeted for nurses and other medical professionals^21^. The echocardiography modules are centered around rheumatic heart disease (RHD) diagnosis but also contain basic cardiac anatomy, a review of the general physics of echocardiography, instructions on how to obtain the major echocardiographic reference views and how to perform echocardiographic sweeps and interpret color Doppler. Following module completion, participants were asked to complete a 20-point competency assessment in the form of an online multiple-choice quiz, hosted in REDCap, and developed for this project. A passing score of 80% was required, with the option of a single repeat of online training/quiz if passing score was not initially achieved.

Part 1, Stage 2: In the second stage, four cardiologists conducted a two-day in-person workshop that reinforced concepts of cardiac anatomy and physiology as well as basic TTE physics and standardized views. Stage 2 training included approximately 8 hours of classroom time and 10 hours of hands-on ultrasound practice. Handheld TTE machines were introduced (General Electric Medical Systems, Milwaukee, Wisconsin, USA). The Vscan is a small pocket-sized device that uses a phased-array transducer (1.7-3.8 MHz) and displays gray scale images and color Doppler images, but lacks spectral Doppler and M-mode capabilities.

Short acquisition and interpretation protocols were taught. The acquisition protocol included parasternal long and short axis, apical 4- and 5-chamber, and limited sub-costal imaging (Appendix, Table 1A). Interpretation focused on visual, semi-quantitative interpretation of most pathological issues (Appendix, Table 1B).

Eight hours of supervised hands-on training was provided for each participant. On day 1, participants practiced image acquisition and interpretation on volunteer patients with structurally and functionally normal hearts. On day two, participants scanned and interpreted echocardiograms of eight volunteer patients with a range of common cardiac pathologies (dilated cardiomyopathy, pericardial effusion, rheumatic heart disease, hypertensive heart failure, and congenital heart defects).

Part 1, Stage 3 consisted of 10 weeks of independent practice with remote mentorship. Participants, on a rotating schedule, performed TTE for patients presenting to LRRH and meeting study inclusion criteria. At the time of acquisition, participants interpreted studies in real-time, and entered their preliminary interpretation, consisting of 9 parameters (Table 1B), in REDCap, an online data entry platform. Studies were uploaded daily to a secure DropBox folder where study investigators could access and interpret images using commercially available software (Vscan Gateway). Images were reviewed within 24 hours by an expert in Uganda or the United States, and feedback on image quality, adherence to the acquisition protocol, and image interpretation were provided thorough REDCap

Participants had varied scanning schedules based on their clinical responsibilities but were asked to log a minimum of 30 studies, chosen pragmatically based on expected number of patients expected to present during the practicum period.

Part 1, Stage 4 consisted of competency assessment offered to participants who achieved the minimum number (30) of practice studies. This final assessment consisted of obtaining and interpreting TTE for 10 standardized patients (a mix of normal patients and those with known pathology) and interpreting ten computer-based patient cases, with stored handheld images for review. Participants were judged both on the quality and completeness of their TTE images (requiring an 80% score to pass) and on their final diagnosis of the patient into one of eight major categories (Table 3), also requiring 80% to pass.
